# Epidemiology and Predictors of NTM Pulmonary Infection in Taiwan - a Retrospective, Five-Year Multicenter Study

**DOI:** 10.1038/s41598-017-16559-z

**Published:** 2017-11-24

**Authors:** Hung-Ling Huang, Meng-Hsuan Cheng, Po-Liang Lu, Chin-Chung Shu, Jann-Yuan Wang, Jann-Tay Wang, Inn-Wen Chong, Li-Na Lee

**Affiliations:** 1 Department of Internal Medicine, Division of Pulmonary and Critical Care Medicine, Kaohsiung Medical University Hospital, Kaohsiung, Taiwan; 2 Departments of Respiratory Therapy, Kaohsiung Medical University Hospital, Kaohsiung, Taiwan; 3 Department of Laboratory Medicine, Kaohsiung Medical University Hospital, Kaohsiung, Taiwan; 4 Graduate Institute of Medicine, College of Medicine, Kaohsiung Medical University, Kaohsiung, Taiwan; 5 Department of Traumatology, National Taiwan University Hospital, Taipei, Taiwan; 6 Department of Internal Medicine, National Taiwan University Hospital, Taipei, Taiwan; 7 Department of Laboratory Medicine, Fu Jen Catholic University Hospital, New Taipei City, Taiwan

## Abstract

Multicenter, longitudinal studies on nontuberculous mycobacteria (NTM) pulmonary infection (PI) are lacking. This study provides a 5-year epidemiological overview of NTM-PI in Taiwan and investigated its predictors. The clinical relevance of each respiratory NTM isolate in six hospitals between 2008 and 2014 was determined according to current guidelines. Recurrent episodes were judged by serial bacteriological results. New episodes of NTM-PI and pulmonary colonization (PC) occurring since 2010 were analyzed. Logistic regression analysis was performed to identify the predictors of NTM-PI. Between 2010 and 2014, the incidence rate of NTM-PI was 46.0 episodes per 100,000 hospital-based patient-years. *Mycobacterium avium intracellulare* complex (MAC) was predominant in Northern Taiwan, whereas MAC and *M. abscessus* were copredominant in Southern Taiwan. Multiple episodes occurred in 9.5% of NTM-PI patients. No female predominance was observed, except for MAC-PI. Previous pulmonary tuberculosis and chronic obstructive pulmonary disease (COPD) were the most common pulmonary comorbidities and independent risk factors for NTM-PI. Other risk factors included *M. kansasii*, *M. abscessus*, and southern Taiwan. Geographical variation of NTM-PI exists in Taiwan. Clinicians should keep a high suspicion on NTM-PI in the risk population. In endemic area of tuberculosis and COPD, there may be no female predominance in NTM-PI.

## Introduction

Pulmonary involvement accounts for 75–94% of all nontuberculous mycobacteria (NTM)-induced human diseases^1,2^. In the past 20 years, a global increase has been reported in both number of clinical isolates and prevalence of NTM^3–5^. The most common species causing NTM pulmonary infection (PI) are *Mycobacterium avium*-*intracellulare* complex (MAC), *M. kansasii*, and *M*. abscessus. However, their relevance ranking varied in different epidemiological reports^3^, which indicates geographical diversity and suggests that single-center data cannot represent the general picture of NTM-PI.

Determining the incidence rate of NTM-PI is challenging because case selection and identification are difficult. Currently, NTM-PI is diagnosed based on composite and complex criteria that were established by the American Thoracic Society and Infectious Disease Society of America (ATS/IDSA) in 2007^2^. Unlike tuberculosis (TB), a public health notification is not mandatory for NTM-PI. Although prospective cohort studies may confirm the exact incidence rate of NTM disease, the long-term follow-up is extremely time-consuming and labor-intensive in large populations. Therefore, most studies have had a single-center, retrospective, and cross-sectional design that determined prevalence rather than incidence rate^1,6–9^. Another limitation of the available reports on NTM-PI is the lack of data on recurrent episodes^10^.

Therefore, this 5-year, longitudinal study investigated the incidence rate of NTM-PI by reviewing the medical records, laboratory data, and chest radiographs of patients with respiratory NTM in six hospitals. We identified the recurrent episodes of NTM-PI and determined the predictors of NTM-PI.

## Results

### Identification of new episodes of NTM-PI and NTM-PC

**Figure 1:**
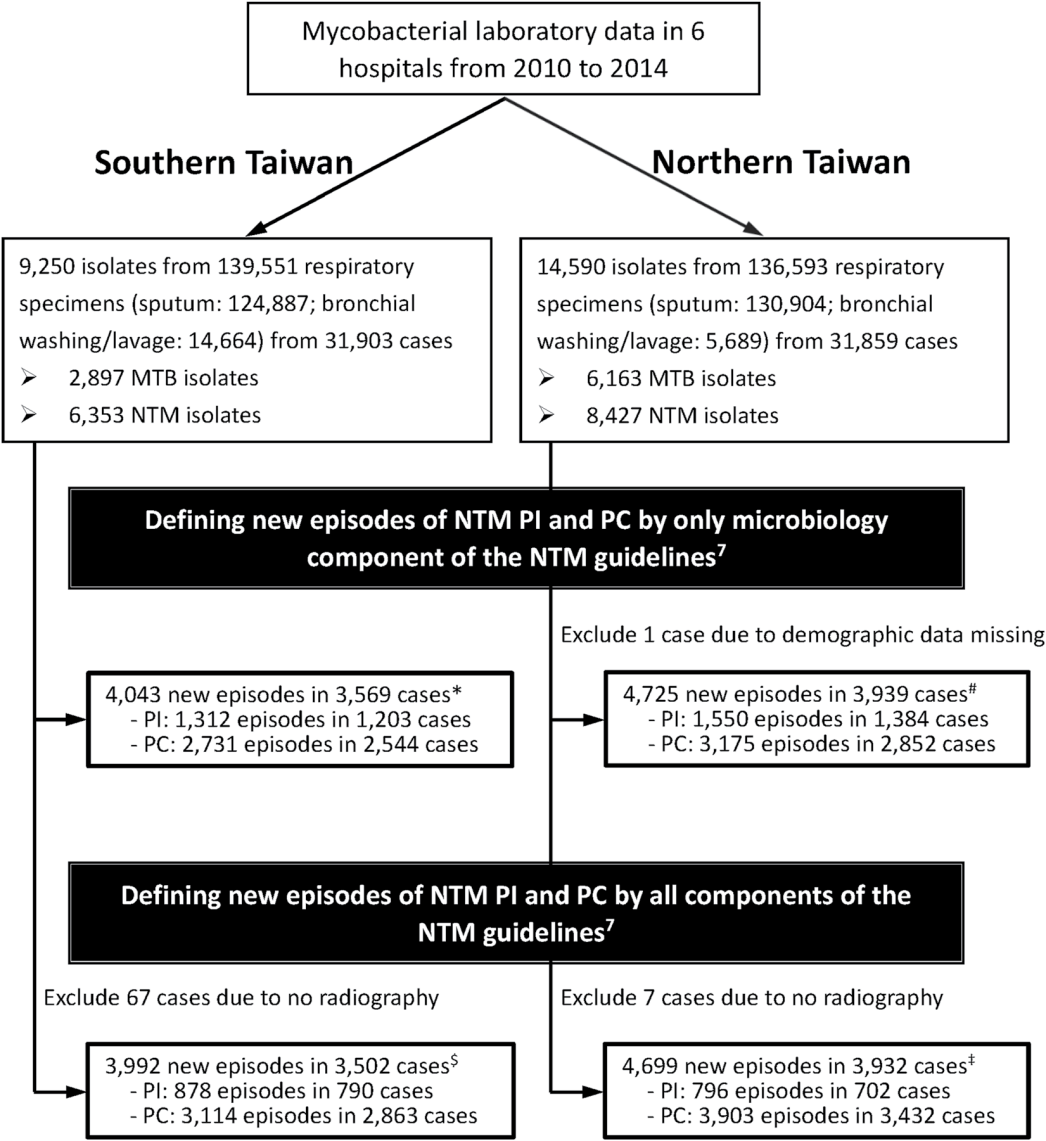
Flowchart of case selection and identification of new episodes of nontuberculous mycobacteria (NTM) pulmonary infection (PI) and pulmonary colonization (PC) in six hospitals (178*, 197^#^, 151^$^, 202^‡^ cases had both NTM-PI and NTM-PC, and were counted in both groups).

Between 2010 and 2014, a total of 1,674 new episodes of NTM-PI in 1,492 cases and 7,017 new episodes of NTM-pulmonary colonization (PC) in 6,295 cases were identified (Fig. 1). Among the 1,492 cases, 141 (9.5%) experienced multiple episodes of NTM-PI; recurrent infection occurred in 36 (25.5%) of them because of same NTM species.

**Figure 2:**
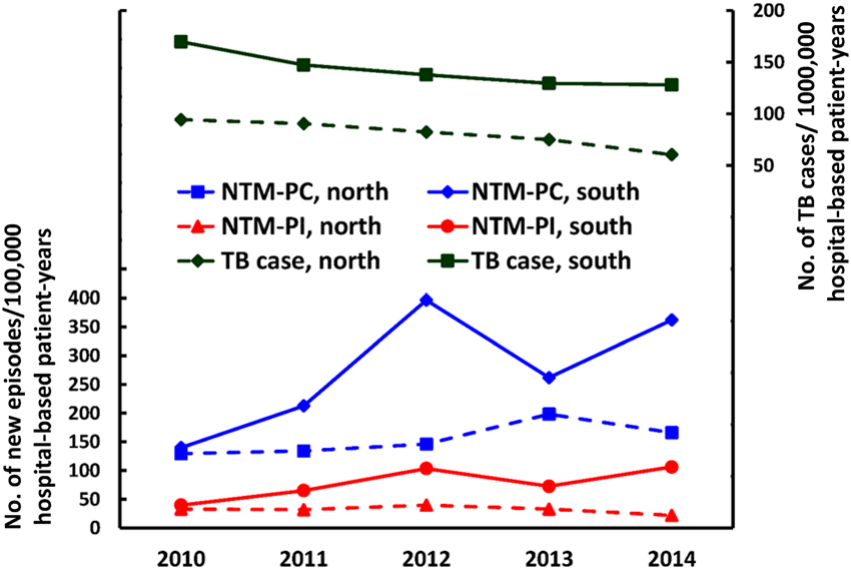
Incidence rate of new episodes of nontuberculous mycobacteria (NTM) pulmonary infection (PI) and pulmonary colonization (PC), as well as incidence of tuberculosis (TB) in northern and southern Taiwan.

The number of respiratory specimens was the lowest in 2010 (n = 40,657) and highest in 2012 (n = 71,771) (Table 1). The number of new infection episodes was stable in Northern Taiwan. However, in Southern Taiwan, the number of new infection episodes peaked in 2014 and that of new *M*. *kansasii* infection gradually increased (Table 1). Between 2010 and 2014, the incidence rate of pulmonary TB gradually declined in the entire cohort (i.e., both Northern and Southern Taiwan; Fig. 2). However, the incidence rate of NTM-PI remained stable and that of NTM-PC gradually increased and reached a plateau after 2012.

**Table 1:** Number of respiratory samples, nontuberculous mycobacteria (NTM) isolates and new infection episodes in six hospitals (three each in northern and southern Taiwan) from 2010 to 2014

	No. of respiratory samples or NTM isolates					No. of NTM-PI episodes				
	2010	2011	2012	2013	2014	2010	2011	2012	2013	2014
**Northern Taiwan**										
Respiratory samples	21,966	23,158	37,254	28,165	26,050	—	—	—	—	—
NTM isolates (Total)	1,193	1,348	2,093	2,054	1,739	158	157	204	169	119
*M. abscessus*	189	257	472	364	395	25	42	34	31	33
MAC	381	488	771	1051	741	60	64	71	84	58
*M. kansasii*	68	102	155	106	161	18	12	19	12	11
Others	555	501	695	533	442	55	39	80	42	17
**Sothern Taiwan**										
Respiratory samples	18,691	20,472	34,517	31,033	34,838	—	—	—	—	—
NTM isolates (Total)	606	968	1,821	1,230	1,728	87	114	230	172	253
*M. abscessus*	147	274	411	318	347	28	46	60	51	58
MAC	157	207	399	241	368	31	43	59	46	61
*M. kansasii*	15	55	147	108	232	3	10	25	17	57
Others	287	432	864	563	781	25	45	86	58	77

Abbreviation: MAC, *Mycobacterium avium-intracellulare* complex.

### Characteristics of new episodes of NTM-PI and NTM-PC

Table 2 shows the clinical characteristics and underlying comorbidities of patients with new episodes of NTM-PI and NTM-PC. Most patients were aged ≥65 years. The male: female ratio was 1.26 and 1.35 in the NTM-PI and NTM-PC groups, respectively. The most common pulmonary comorbidities were previous pulmonary TB (22.1%) and chronic obstructive pulmonary disease (COPD) (19.8%), whereas the most common systemic illnesses were malignancy (8.9%) and diabetes mellitus (DM) (8.3%). In the NTM-PI group, the most common symptom was cough (75.5%) and sputum (74.5%).

**Table 2:** Clinical characteristics, radiographic findings and laboratory data of new episodes of nontuberculous mycobacterial (NTM) pulmonary infection and colonization in six hospitals.

Characteristics	New Infection (1,674 episodes)	New Colonization (7,016 episodes)	*p*-value
**Age group**			
Age < 25	11 (0.7%)	129 (1.8%)	0.001
Age 25~44	110 (6.6%)	684 (9.7%)	<0.001
Age 45~64	560 (33.5%)	2,202 (31.4%)	0.103
Age >=65	993 (59.3%)	4,001 (57.0%)	0.088
**Gender**			
Male	932 (55.7%)	4027 (57.4%)	0.200
**Comorbidity**			
History of pulmonary TB	425 (25.4%)	1495 (21.3%)	<0.001
COPD	392 (23.4%)	1328 (18.9%)	<0.001
Bronchiectasis	261 (15.6%)	535 (7.6%)	<0.001
Interstitial lung disease	101 (6.0%)	261 (3.7%)	<0.001
Asthma	87 (5.2%)	312 (4.4%)	0.304
Pneumoconiosis	13 (0.8%)	36 (0.5%)	0.199
Cancer	154 (9.2%)	623 (8.9%)	0.680
Diabetes mellitus	121 (7.2%)	602 (8.6%)	0.072
Congestive heart failure	100 (6.0%)	323 (4.6%)	0.020
Autoimmune disease	63 (3.7%)	140 (2.0%)	<0.001
HIV infection	48 (2.9%)	134 (1.9%)	0.015
Liver cirrhosis	29 (1.7%)	121 (1.7%)	0.983
Transplant	15 (0.9%)	46 (0.7%)	0.292
Chronic kidney disease	13 (0.8%)	50 (0.7%)	0.782
Steroid user	108 (6.5%)	331 (4.7%)	0.004
**NTM species**			
MAC	577 (34.4%)	1818 (26.0%)	<0.001
*M. abscessus*	408 (24.3%)	1059 (15.1%)	<0.001
*M. fortuitum*	164 (9.8%)	1266 (18.0%)	<0.001
*M. kansasii*	184 (11.0%)	403 (5.7%)	<0.001
*M. gordonae*	78 (4.7%)	826 (11.7%)	<0.001
Other NTM species	263 (15.7%)	1644 (23.4%)	<0.001
**CXR pattern**			
Fibocavitary	517 (30.9%)		
Nodular bronchiectatic	1157 (69.1%)		
**CXR extent**			
Focal	475 (28.4%)		
Multifocal	1199 (71.6%)		
Blood tests*			
Hemoglobin <12 g/dL	432 (39.7%)	1492 (39.2%)	0.782
Platelet count <140 K/uL	140 (12.9%)	574 (15.1%)	0.067
Leukocyte <4000 or >10500/uL	299 (27.5%)	986(25.9%)	0.304
Segment >70%	456 (54.9%)	1440 (48.5%)	0.004
C-reactive protein >5 mg/L	522 (70.6%)	1640 (67.7%)	0.135
AST >40 U/L	184 (18.3%)	616 (17.7%)	0.678
ALT >40 U/L	138 (13.1%)	522 (14.0%)	0.454
Total bilirubin >1.0 mg/dL	146 (18.2%)	565 (20.9%)	0.096
Creatinine >1.4 mg/dL	125 (11.4%)	511 (13.2%)	0.099

Data are number (%).

Abbreviation: ALT, alanine transaminase; AST, aspartate transaminase; COPD, chronic obstructive pulmonary disease; MAC, *Mycobacterium avium*-*intracellulare* complex; TB, tuberculosis.

*Data are the percentage of episodes with the characteristics among all tested episodes.

Compared with patients with NTM-PC, those with NTM-PI were older and were more likely to have structural lung diseases (including a history of pulmonary TB, COPD, bronchiectasis, or interstitial lung disease [ILD]), congestive heart failure, an autoimmune disease, HIV infection, and steroid usage (Table 2).

The female sex was not associated with NTM-PI in the entire cohort, and in different NTM species except for MAC (Table 2, Table S2 and Figure S2). Moreover, compared with men, women had a higher prevalence of bronchiectasis (19.7% vs. 12.3%, *p* < 0.001) and ILD (7.8% vs. 4.6%, *p* = 0.007), and a lower prevalence of previous pulmonary TB (21.6% vs. 28.4%, *p* = 0.001), COPD (18.9% vs. 27.0%, *p* < 0.001), and pneumoconiosis (0% vs. 1.4%, *p* < 0.001; Table S3).

MAC, *M. abscessus*, and *M. kansasii* were the most common species of NTM-PI in either the entire cohort (Table 2) or in all age groups (Figure S3). Among the 878 new episodes of NTM-PI in Southern Taiwan, the most common species were *M. abscessus* (n = 243, 27.7%) and MAC (n = 240, 27.3%). However, in Northern Taiwan, only MAC was predominant (n = 337, 42.3%).

The FC and NB patterns were the major radiographic findings in 517 (30.9%) and 1157 (69.1%) episodes of NTM-PI, respectively (Table 2). The proportion of men was higher in the FC group than in the NB group (65.4% vs. 51.3%, *p* < 0.001). A history of TB was more common in the FC group than in the NB group (33.5% vs. 21.8%, *p* < 0.001). Lesions with an FC pattern were more common in *M. kansasii* (42.4%) infection than MAC (28.6%, *p* < 0.001) or *M. abscessus* (26.2%, *p* < 0.001) infections (Table S4). Multifocal involvement was most common in *M. kansasii* infection (78.8%), followed by MAC (75.4%) and *M. abscessus* (69.6%) infections.

Blood examination (Table 2) mostly revealed similarities between the NTM-PI and NTM-PC groups, although neutrocytosis was more common in the NTM-PI group (54.9% vs. 48.5%, *p* = 0.004). Elevation in the serum C-reactive protein (CRP) level, anemia (hemoglobin < 12 g/dL) and thrombocytopenia (platelet < 140 k/µL) were observed in 70.6%, 39.7% and 12.9% of patients, respectively, in the NTM-PI group.

### Predictors of NTM-PI

Multivariate logistic regression analysis on the new episodes of NTM-PI and NTM-PC revealed that NTM isolates in Southern Taiwan were more likely to cause pulmonary infection than those in Northern Taiwan (Table 3). An age of 25–65 years was a protector of NTM-PI; other independent predictors for NTM-PI included the underlying pulmonary diseases (history of pulmonary TB, COPD, and bronchiectasis) and systemic comorbidities (autoimmune disease and HIV infection). NTM-PI was more commonly caused by MAC (OR: 2.34 [2.03–2.70]), *M. abcessus* (OR: 2.92 [2.50–3.42]), or *M. kansasii* (OR: 3.41 [2.78–4.19]).

**Table 3:** Independent risk factors of new episode of pulmonary infection by nontuberculous mycobacteria in multivariate logistic regression analysis.

Characteristics	Odds Ratio (95% CI)	*p*-value
Location (southern vs. northern Taiwan)	1.73 (1.54–1.94)	<0.001
Age between 25~45 (yes vs. no)	0.36 (0.19–0.67)	0.001
Age between 45~65 (yes vs. no)	0.66 (0.54–0.82)	<0.001
Previous history of tuberculosis (yes vs. no)	1.31 (1.15–1.49)	<0.001
Chronic obstructive pulmonary disease (yes vs. no)	1.17 (1.01–1.34)	0.032
Bronchiectasis (yes vs. no)	2.14 (1.80–2.54)	<0.001
Autoimmune (yes vs. no)	1.79 (1.30–2.46)	0.001
Acquired immunodeficiency syndrome (yes vs. no)	1.51 (1.06–2.16)	0.022
*M. avium*-*intracellulare* complex (yes vs. no)	2.34 (2.03–2.70)	<0.001
*M. abscessus* (yes vs. no)	2.92 (2.50–3.42)	<0.001
*M. kansasii* (yes vs. no)	3.41 (2.78–4.19)	<0.001

All variables in Table 2 except radiographic pattern and extent were included in multivariate logistic regression analysis.

Predictors of NTM-PI were further investigated by ‘patients’, instead of ‘episodes’. Among the 7434 patients, 809 had multiple episodes at different time points. For them only the first episode was included. Another 178 had NTM isolates of different species at the same time and was excluded from this analysis. For the 7256 patients analyzed, the independent predictors of NTM-PI were very similar as those identified in the analysis done by ‘episodes’ (Table S5).

## Discussion

This first multicenter NTM study conducted in Taiwan yielded several important findings. First, NTM-PI is common, with an average incidence rate of 46.0 episodes per 100,000 hospital-based patient-years between 2010 and 2014. In total, 9.5% of patients with NTM-PI experienced multiple episodes, and recurrent infections in 24.8% of them were caused by the same NTM species. In Southern Taiwan, the incidence rate of NTM-PI increased slightly over 5 years, particularly that caused by *M. kansasii*. Second, NTM-PI occurs equally in men and women in Taiwan, and is more likely to occur in the elderly, patients with structural lung diseases, and those living in southern Taiwan. Third, *M. abscessus* and MAC are copredominant in Southern Taiwan, whereas only MAC is dominant in Northern Taiwan.

The diagnosis of NTM-PI based on the ATS/IDSA guidelines requires a detailed review of extensive medical records, which is extremely time-consuming and labor-intensive^2^. Therefore, in many studies, clinical symptoms and radiographic findings were omitted and only the microbiological criteria were used as the diagnostic standard of NTM-PI^3,11–13^, sometimes with modifications^14,15^. According to present study, among those who fulfilled the microbiological component of the ATS diagnostic criteria for NTM-PI, 41.5% had no typical radiographic findings or no compatible clinical symptoms. Therefore, they were not initially deemed to be NTM-PI by primary care physicians. Furthermore, most studies have determined the prevalence rate, rather than the incidence rate^1,6–9^.

Because a large proportion of patients with NTM isolates have underlying structural lung diseases, clinical symptoms and radiographic abnormalities may indeed result from structural lung diseases rather than from NTM-PI. Diagnosing NTM-PI is difficult unless patients were longitudinally followed.

Previous studies revealed female predominance in NTM-PI^2,16^. Though several hypotheses have been proposed^1,17–20^, the underlying mechanism remains controversial. One possible explanation for the discrepancies in female predominance in this study is that TB is endemic in Taiwan, with a male: female ratio of 2.2–2.3^21^. COPD is also more prevalent in men (male: female ratio: 1.9)^22^. Because both old pulmonary TB and COPD were the most common underlying pulmonary comorbidities in this study, the male predominance in the two diseases could offset the effects of female sex on the risk of NTM-PI.

In concordance with previous reports, the current study revealed that the incidence rate of NTM-PI increased with age^13,23^. This may reflect the accumulation of structural changes in lung parenchyma during aging. Thus, with the global trend of population aging^24^, we can expect a progressive increase in the prevalence of NTM diseases and their consequent medical expenses in the future.

The species distribution of NTM-PI varies worldwide; however, the exact mechanism remains unclear. MAC and *M. kansasii* are the most common species in most parts of the United States, Australia (Queensland), South Africa, and Japan^2,11,14^. In Singapore, southeastern United States (from Florida to Texas), Okinawa, and South Australia, rapidly growing mycobacteria (RGM), particularly *M. abscessus*, are more predominant^25–27^. In a recent meta-analysis of 105 publications from China, the ratio of MAC in NTM isolates was reported to increase with the latitude, whereas that of RGM isolates showed a contrasting trend^5^. Taiwan lies on the Tropic of Cancer; thus, the climate of Northern and Central Taiwan is subtropical, whereas that of Southern Taiwan is tropical. This might partially explain the MAC predominance in Northern Taiwan and the increased proportion of *M. abscessus* in Southern Taiwan^13,15^.

The increase of the incidence rate of *M. kansasii*-PI in southern Taiwan is a serious concern for its high clinical relevance^28^. The industrial activities, such as gold mining and iron manufacturing, may be associated with *M*. *kansasii* infection, particularly in cases of previous TB scarring or silicosis^14,29,30^. Southern Taiwan is famous for its iron and steel industries, as well as shipbuilding. Industrial water pollution and chlorination for disinfection may further favor the emergence of *M. kansasii* because of its natural resistance to disinfectants, acid, and heat, and its affinity for pipe surfaces^31,32^. The intense air pollution caused by heavy doses of particulates may also aid mycobacterial invasion and infection^33^, and the relatively wetter and hotter climate in Southern Taiwan might facilitate the survival of *M. kansasii* ^27,34^. These environmental factors might also explain the finding that southern Taiwan was an independent risk factor of NTM-PI.

The study has some limitations. First, it is a retrospective study without a standard protocol. Data on some critical factors, such as lifestyle, living environment, smoking history, occupation, and body mass index, are lacking. Second, the NTM isolates were not identified to the subspecies level, which have been revealed associated with different clinical manifestations^33^. Third, because data from only two medical centers and their branch hospitals were analyzed in this study, the findings may not entirely represent the incidence of NTM-PI in Taiwan. Forth, even we try to identify active lung lesions by reviewing clinical symptoms and serial chest images for the 141 cases suffering from ≥2 episodes of NTM-PI, the later episodes may be misjudged due to the radiographic sequelae of previous NTM-PI episodes. Lastly, due to lacks of results of antibiotic susceptibility test, it was not possible to predict the clinical effectiveness of specific antimicrobials. A prospective study with comprehensive case information and a standardized follow-up protocol in more hospitals in different areas of Taiwan is recommended to confirm the present findings.

In conclusion, this is the first multicenter, longitudinal NTM epidemiological report to show geographical diversity in NTM distribution and describe patient characteristics and the recurrent nature of NTM-PI. The findings remind clinicians that NTM-PI is common in Taiwan (46.0 episodes per 100,000 hospital-based patient-years), especially in elderly patients with structural lung disease in southern Taiwan. There may be no female predominance in endemic area of TB and COPD. NTM-PI should not be diagnosed solely based on the microbiological criteria of the ATS/IDSA guidelines because the overestimation of disease incidence is likely.

## Methods

### Study population

This retrospective study was conducted in six hospitals, namely a 2600-bed medical center (Taipei) and its two branch hospitals (Taipei and Hsinchu) in Northern Taiwan, and a 2665-bed medical center (Kaohsiung) and its two branch hospitals (both in Kaohsiung) in Southern Taiwan. This study was approved by the institutional ethic committees of both hospitals (NTUH REC 201508017RIND and KMUHIRB-SV[I]-2015200266).

Between 2008 and 2014, respiratory specimens were retrieved from the mycobacteriological databases of these six hospitals. NTM were classified into the following groups: *M. kansasii*, MAC, *M. abscessus*, *M. fortuitum*, *M. gordonae*, and other NTM species (see the supplementary file for mycobacteriology methodology). The clinical relevance of each NTM isolate was determined according to the ATS/IDSA guidelines^2^. First, patients with NTM isolates who fulfilled the microbiological criteria were identified, and those with typical radiographic findings suggestive of NTM-PI (fibrocavitary [FC] or nodular bronchiectatic [NB]) were further selected (detailed methodology in the supplementary file and Table S1). Subsequently, clinical symptoms, medical history, and other laboratory data were reviewed to exclude the diagnoses of diseases other than NTM-PI (Fig. 1). Clinical symptoms included cough, sputum, hemoptysis, chest tightness, dyspnea, fever, weight loss, night fever, and poor appetite. NTM-pulmonary colonization (PC) was considered if not fulfilling all of the aforementioned factors.

To determine the hospital-based incidence rate and to record the recurrent nature, new episodes of NTM-PI or NTM-PC occurring between 2010 and 2014 were used as the numerator, and the denominator was hospital-based patient-years, for which one patient with any outpatient or inpatient visit within one specific year was counted as 1 patient-year. A new episode of NTM-PI or NTM-PC was considered if either of the following conditions was established: (1) no culture was performed or no same NTM species were isolated in the previous 2 years, or (2) at least two respiratory samples were cultured and no isolates of the same NTM species were obtained in the previous year.

### Data collection

Patient demographics and underlying comorbidities before obtaining each NTM isolate were retrieved. The comorbidities were categorized as pulmonary (e.g., COPD, asthma, previous pulmonary TB, bronchiectasis, pneumoconiosis, and ILD), and extrapulmonary (e.g., DM, autoimmune disease, chronic kidney disease [CKD], post-transplantation, HIV infection, congestive heart failure [CHF], and steroid usage). Furthermore, the TB registry databases of the Taiwan Centers for Disease Control were used to retrieve the history of pulmonary TB. Laboratory data included hemogram (leukocyte count and percentage of segment, hemoglobin level, platelet count) and serum biochemistry (creatinine, aspartate transaminase [AST], alanine transaminase [ALT], total bilirubin, and CRP) within 3 months of each sample yielding NTM isolate.

### Statistical analysis

All data were expressed as numbers (percentage) or means ± standard deviations. Intergroup differences were analyzed using independent sample *t* tests for continuous variables and chi-square tests for categorical variables. Multivariate logistic regression analysis was performed to identify the independent risk factors for NTM-PI among all episodes of NTM-PI and NTM-PC. Statistical significance was set at *p* < 0.05. All statistical analyses were performed using SAS (Version 9.3; SAS Institute Inc., Cary, NC, USA).

## Electronic supplementary material

Supplementary File
