# Supplementary material for: Epidemiology and Predictors of NTM Pulmonary Infection in Taiwan - a Retrospective, Five-Year Multicenter Study
Source: Sci Rep. 2017 Nov 24;7:16300. doi: 10.1038/s41598-017-16559-z (PMC5701172; doi:10.1038/s41598-017-16559-z)
Supplement: Supplementary file 1 — Supplementary File [file 41598_2017_16559_MOESM1_ESM.doc]

**Epidemiology and Predictors of NTM Pulmonary Infection in Taiwan - a Retrospective, Five-Year Multicenter Study**

Hung-Ling Huang,^1^ Meng-Hsuan Cheng,^1,4^ Po-Liang Lu,^3,4^ Chin-Chung Shu,^5^ Jann-Yuan Wang,^6^ Jann-Tay Wang,^6^ Inn-Wen Chong,^1,2,4^ Li-Na Lee^7^

**Supplementary File**

**Laboratory procedures for mycobacterial study of respiratory specimens**

All respiratory specimens sent for mycobacterial culture were processed as previously described.^1^ Culturing was performed using Middlebrook 7H11 selective agar with antimicrobials (Remel Inc., Lexena, KS, USA) and the fluorometric BACTEC technique (BACTEC MGIT 960 system; Becton–Dickinson Diagnostic Instrument Systems, Sparks, MD, USA), and mycobacterial species were identified through biochemical testing.^2^ The quality control assessment of the mycobacterial laboratories was periodically performed by the National Reference Laboratory of the Centers for Disease Control of Taiwan.^3^

**Chest radiograph interpretation**

Chest radiography performed within 1 month of each specimen detected as nontuberculous mycobacterium was randomly reviewed by one of the nine pulmonologists who were blinded to the clinical information. A standard format was used to characterize the pattern (FC, NB, or other) and extent (multifocal or focal) of radiographic abnormalities (Figure S1). Before interpretation, two rounds of consensus testing, each comprising 20 radiographies, were conducted. The kappa values of the pattern and extent were 0.243 and 0.388, respectively, in the first round, and then increased to 0.602 and 0.928, respectively, in the second round (Table S1).

**Reference**

1. Lee, M.R.*, et al.* Factors associated with subsequent nontuberculous mycobacterial lung disease in patients with a single sputum isolate on initial examination. *Clin. Microbiol. Infect.* **21**, 250 e251-257 (2015).

2. Brown-Elliott, B.A. & Wallace, R.J., Jr. Clinical and taxonomic status of pathogenic nonpigmented or late-pigmenting rapidly growing mycobacteria. *Clin. Microbiol. Rev.* **15**, 716-746 (2002).

3. Wu, M.H., Chiang, C.Y., Deng, Y.M., Wang, T.F. & Jou, R. Proficiency of drug susceptibility testing for Mycobacterium tuberculosis in Taiwan, 2007-2011. *Int. J. Tuberc. Lung Dis.* **17**, 113-119 (2013).

**Supplementary Figure S1.** Typical radiographic findings suggestive of nontuberculous mycobacteria pulmonary infection (S1A: focal fibrocavitary; S1B: focal nodular bronchiectatic; S1C: multifocal fibrocavitary; S1D: multifocal nodular bronchiectatic)


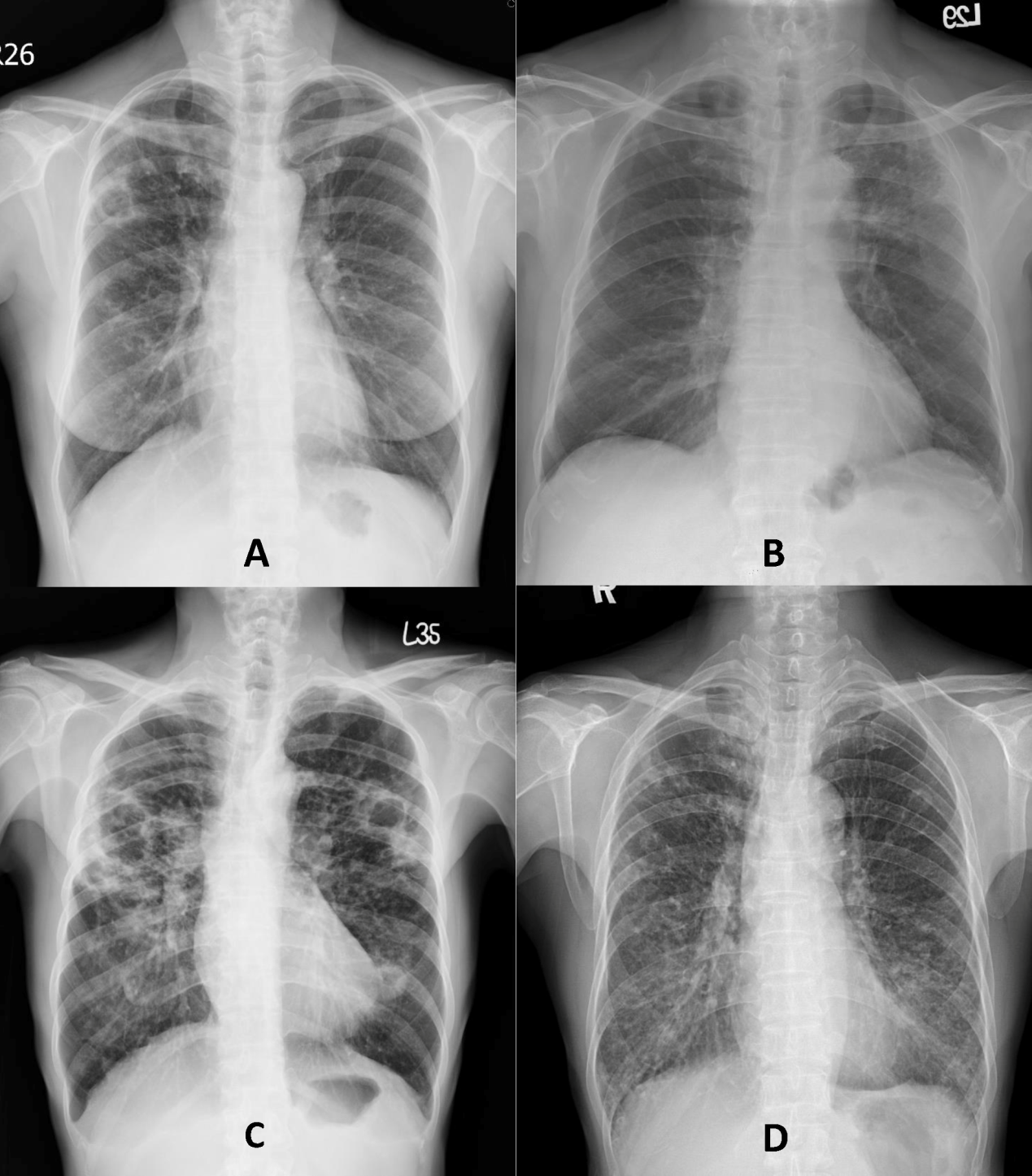


**Supplementary Figure S2.** Percentage of episodes of nontuberculous mycobacteria pulmonary infection (PI) and colonization (PC) in women and men (numbers inside each bar represents number of PI episodes) (MAC: *Mycobacterium avium*-*intracellular* complex)


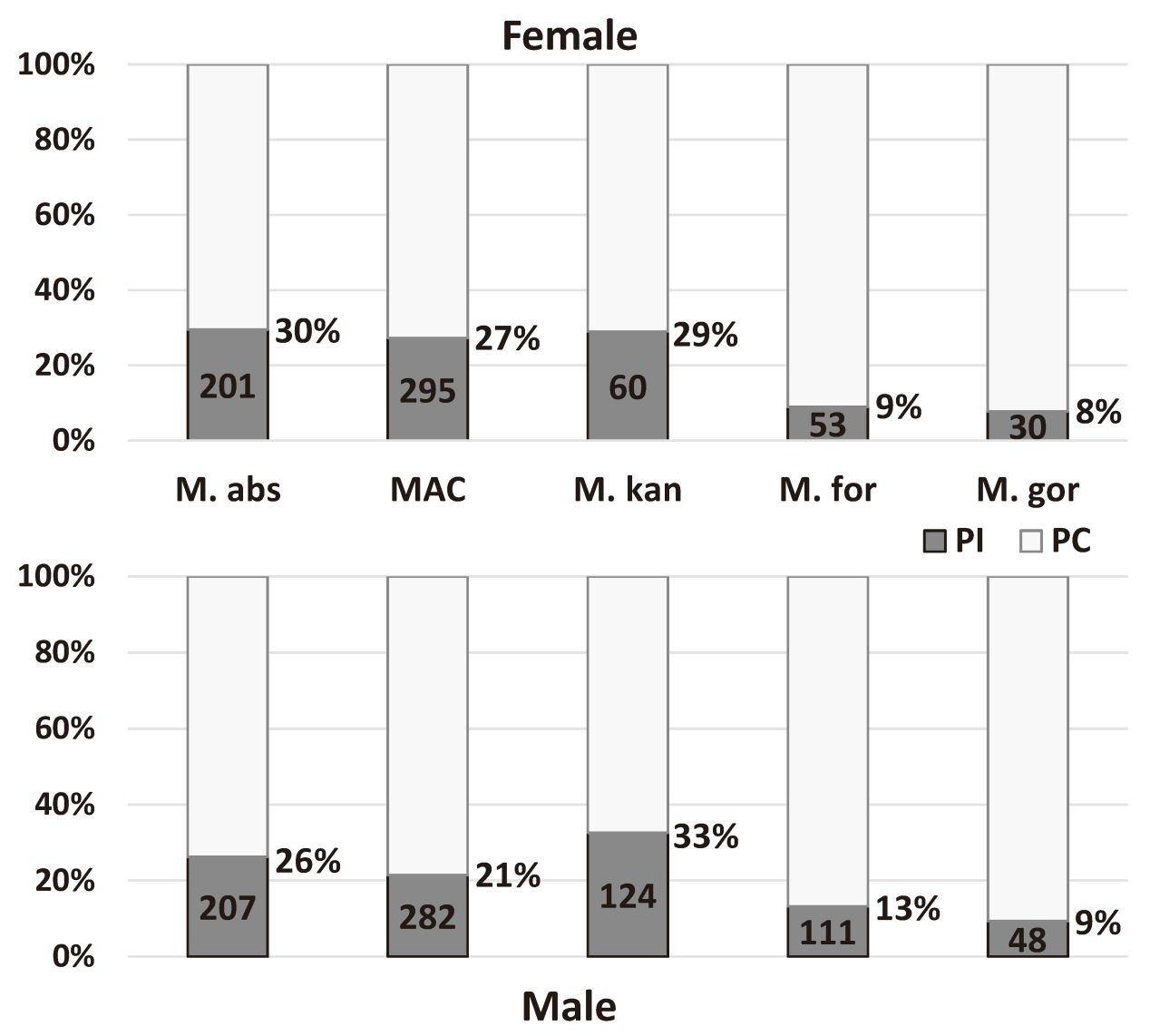


**Supplementary Figure S3.** Numbers of new episodes of nontuberculous mycobacteria pulmonary infection (PI) and colonization (PC) in different age groups in southern and northern Taiwan (MAC: *Mycobacterium avium*-*intracellulare* complex)


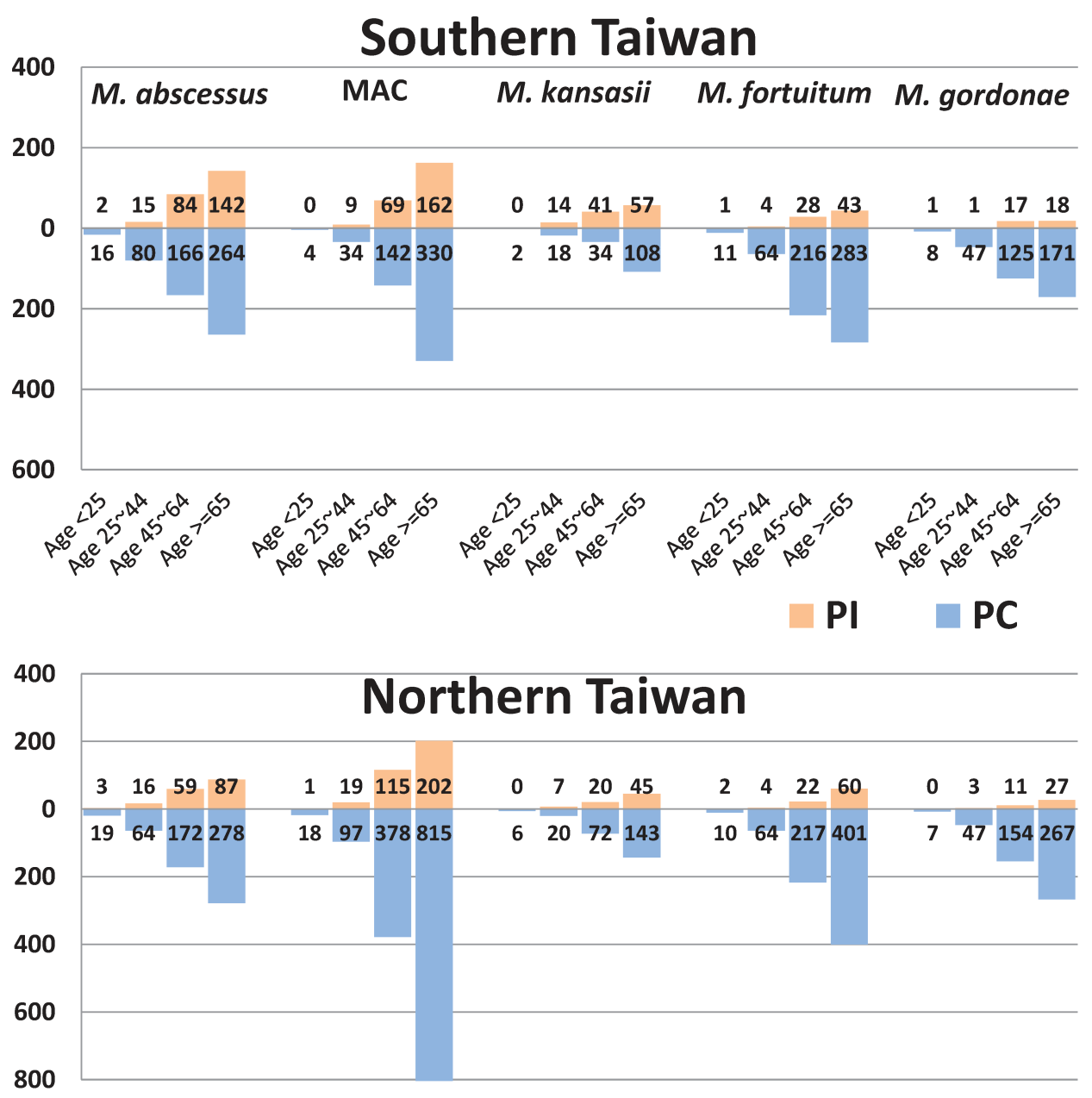


**Supplementary Table S1.** Inter-rater (n=9) agreement in consensus training for chest radiography (CXR) reading

| **Chest radiography** | **Pattern** | | |  | **Extent** | |
| --- | --- | --- | --- | --- | --- | --- |
|  | **NB** | **FC** | **Other** |  | **Focal** | **Multifocal** |
| **First round** |  |  |  |  |  |  |
| 1 | 5 | 4 | 0 |  | 1 | 8 |
| 2 | 6 | 2 | 1 |  | 1 | 8 |
| 3 | 7 | 1 | 1 |  | 4 | 4 |
| 4 | 8 | 0 | 1 |  | 5 | 4 |
| 5 | 4 | 1 | 4 |  | 3 | 5 |
| 6 | 6 | 3 | 0 |  | 0 | 9 |
| 7 | 6 | 1 | 2 |  | 4 | 4 |
| 8 | 0 | 7 | 2 |  | 8 | 1 |
| 9 | 9 | 0 | 0 |  | 0 | 9 |
| 10 | 7 | 2 | 0 |  | 5 | 4 |
| 11 | 2 | 7 | 0 |  | 1 | 8 |
| 12 | 9 | 0 | 0 |  | 6 | 3 |
| 13 | 8 | 0 | 1 |  | 0 | 9 |
| 14 | 3 | 1 | 5 |  | 1 | 7 |
| 15 | 8 | 1 | 0 |  | 0 | 9 |
| 16 | 2 | 6 | 1 |  | 1 | 8 |
| 17 | 8 | 1 | 0 |  | 2 | 7 |
| 18 | 3 | 1 | 5 |  | 5 | 3 |
| 19 | 6 | 1 | 2 |  | 7 | 1 |
| 20 | 8 | 1 | 0 |  | 0 | 9 |
| **Kappa value** | **0.243** | | |  | **0.388** | |
|  |  |  |  |  |  |  |
| **Second round** |  |  |  |  |  |  |
| 21 | 9 | 0 | 0 |  | 0 | 9 |
| 22 | 0 | 7 | 2 |  | 9 | 0 |
| 23 | 0 | 9 | 0 |  | 9 | 0 |
| 24 | 2 | 7 | 0 |  | 1 | 8 |
| 25 | 0 | 9 | 0 |  | 0 | 9 |
| 26 | 1 | 0 | 8 |  | 9 | 0 |
| 27 | 3 | 6 | 0 |  | 1 | 8 |
| 28 | 8 | 0 | 1 |  | 0 | 9 |
| 29 | 9 | 0 | 0 |  | 0 | 9 |
| 30 | 9 | 0 | 0 |  | 0 | 9 |
| 31 | 1 | 8 | 0 |  | 9 | 0 |
| 32 | 7 | 1 | 1 |  | 0 | 9 |
| 33 | 1 | 7 | 1 |  | 9 | 0 |
| 34 | 0 | 9 | 0 |  | 9 | 0 |
| 35 | 8 | 0 | 1 |  | 0 | 9 |
| 36 | 7 | 0 | 2 |  | 0 | 9 |
| 37 | 2 | 7 | 0 |  | 0 | 9 |
| 38 | 0 | 8 | 1 |  | 9 | 0 |
| 39 | 8 | 1 | 0 |  | 0 | 9 |
| 40 | 1 | 7 | 1 |  | 1 | 8 |
| **Kappa value** | **0.602** | | |  | **0.928** | |

FC: fibrocavitary; NB: nodular bronchiectatic;

Inter-rater agreement was calculated by using Fleiss’ Kappa value (Fleiss JL. Measuring nominal scale agreement among many raters. Psychological Bulletin. 1971;76:378-82).

**Supplementary Table S2A.** Clinical characteristics of new episodes of nontuberculous mycobacterial (NTM) pulmonary infection and colonization in southern Taiwan

| **Characteristics** | **New Infection**  **(878 episodes)** | **New Colonization**  **(3,114 episodes)** | **P-value** |
| --- | --- | --- | --- |
| **Age group** |  |  |  |
| Age <25 | 4 (0.5%) | 58 (1.9%) | 0.006 |
| Age 25~44 | 55 (6.3%) | 341 (11.0%) | <0.001 |
| Age 45~64 | 297 (33.8%) | 994 (31.9%) | 0.286 |
| Age >=65 | 522 (59.5%) | 1721 (55.3%) | 0.027 |
| **Gender** |  |  |  |
| Male | 488 (55.6%) | 1753 (56.3%) | 0.707 |
| **Comorbidity** |  |  |  |
| History of pulmonary TB | 222 (25.3%) | 620 (19.9%) | <0.001 |
| COPD | 137 (15.6%) | 559 (18.0%) | 0.106 |
| Bronchiectasis | 80 (9.1%) | 119 (3.8%) | <0.001 |
| Interstitial lung disease | 42 (4.8%) | 135 (4.3%) | 0.547 |
| Asthma | 24 (2.7%) | 104 (3.3%) | 0.369 |
| Pneumoconiosis | 1 (0.1%) | 6 (0.2%) | 0.626 |
| Cancer | 34 (3.9%) | 89 (2.9%) | 0.126 |
| Diabetes mellitus | 27 (3.0%) | 169 (5.4%) | 0.005 |
| Congestive heart failure | 29 (3.3%) | 99 (3.2%) | 0.854 |
| Autoimmune disease | 20 (2.3%) | 30 (1.0%) | 0.003 |
| HIV infection | 0 (0.0%) | 0 (0.0%) | -- |
| Liver cirrhosis | 9 (1.0%) | 24 (0.8%) | 0.464 |
| Transplant | 0 (0.0%) | 0 (0.0%) | -- |
| Chronic kidney disease | 4 (0.5%) | 18 (0.6%) | 0.666 |
| Steroid user | 49 (5.6%) | 143 (4.6%) | 0.227 |
| **NTM species** |  |  |  |
| MAC | 240 (27.3%) | 510 (16.4%) | <0.001 |
| *M. abscessus* | 243 (27.7%) | 526 (16.9%) | <0.001 |
| *M. fortuitum* | 76 (8.7%) | 574 (18.4%) | <0.001 |
| *M. kansasii* | 112 (12.8%) | 162 (5.2%) | <0.001 |
| *M. gordonae* | 37 (4.2%) | 351 (11.3%) | <0.001 |
| Other NTM species | 170 (19.4%) | 991 (31.8%) | <0.001 |

Data are number (%).

Abbreviation: COPD, chronic obstructive pulmonary disease; MAC, *Mycobacterium avium*-*intracellulare* complex; TB, tuberculosis;

**Supplementary Table S2B.** Clinical characteristics of new episodes of nontuberculous mycobacterial (NTM) pulmonary infection and colonization in northern Taiwan

| **Characteristics** | **New Infection**  **(796 episodes)** | **New Colonization**  **(3,902 episodes)** | **P-value** |
| --- | --- | --- | --- |
| **Age group** |  |  |  |
| Age <25 | 7 (0.9%) | 71 (1.8%) | 0.064 |
| Age 25~44 | 55 (6.9%) | 343 (8.8%) | 0.084 |
| Age 45~64 | 262 (32.9%) | 1209 (31.0%) | 0.282 |
| Age >=65 | 472 (59.3%) | 2279 (58.4%) | 0.637 |
| **Gender** |  |  |  |
| Male | 444 (55.8%) | 2274 (58.3%) | 0.196 |
| **Comorbidity** |  |  |  |
| History of pulmonary TB | 202 (25.4%) | 876 (22.4%) | 0.073 |
| COPD | 255 (32.0%) | 769 (19.7%) | <0.001 |
| Bronchiectasis | 181 (22.7%) | 416 (10.7%) | <0.001 |
| Interstitial lung disease | 59 (7.4%) | 126 (3.2%) | <0.001 |
| Asthma | 63 (7.9%) | 208 (5.3%) | 0.005 |
| Pneumoconiosis | 12 (1.5%) | 30 (0.8%) | 0.048 |
| Cancer | 120 (15.1%) | 534 (13.7%) | 0.301 |
| Diabetes mellitus | 94 (11.8%) | 433 (11.1%) | 0.560 |
| Congestive heart failure | 71 (8.9%) | 224 (5.7%) | <0.001 |
| Autoimmune disease | 43 (5.4%) | 110 (2.8%) | <0.001 |
| HIV infection | 48 (6.0%) | 134 (3.4%) | <0.001 |
| Liver cirrhosis | 20 (2.5%) | 97 (2.5%) | 0.964 |
| Transplant | 15 (1.9%) | 46 (1.2%) | 0.125 |
| Chronic kidney disease | 9 (1.1%) | 32 (0.8%) | 0.435 |
| Steroid user | 59 (7.4%) | 188 (4.8%) | 0.003 |
| **NTM species** |  |  |  |
| MAC | 337 (42.3%) | 1308 (33.5%) | <0.001 |
| *M. abscessus* | 165 (20.7%) | 533 (13.7%) | <0.001 |
| *M. fortuitum* | 88 (11.1%) | 694 (17.8%) | <0.001 |
| *M. kansasii* | 72 (9.0%) | 241 (6.2%) | 0.005 |
| *M. gordonae* | 41 (5.2%) | 475 (12.2%) | <0.001 |
| Other NTM species | 93 (11.7%) | 653 (16.7%) | <0.001 |

Data are number (%).

Abbreviation: COPD, chronic obstructive pulmonary disease; MAC, *Mycobacterium avium-intracellulare* complex; TB, tuberculosis;

**Supplementary Table S2C.** Clinical characteristics of new episodes of *Mycobacterium avium*-*intracellulare* complex (MAC) pulmonary infection and colonization in six hospitals

| **Characteristics** | **New Infection**  **(577 episodes)** | **New Colonization (1,818 episodes)** | **P-value** |
| --- | --- | --- | --- |
| **Age group** |  |  |  |
| Age <25 | 1 (0.17%) | 22 (1.2%) | 0.056 |
| Age 25~44 | 28 (4.9%) | 131 (7.2%) | 0.046 |
| Age 45~64 | 184 (32.0%) | 520 (28.7%) | 0.119 |
| Age >=65 | 364 (63.1%) | 1145 (63.2%) | 0.973 |
| **Gender** |  |  |  |
| Male | 282 (48.9%) | 1030 (56.8%) | 0.001 |
| **Comorbidity** |  |  |  |
| History of pulmonary TB | 128 (22.2%) | 412 (22.7%) | 0.865 |
| COPD | 169 (29.2%) | 400 (22.1%) | <0.001 |
| Bronchiectasis | 132 (23.0%) | 193 (10.7%) | <0.001 |
| Interstitial lung disease | 50 (8.7%) | 87 (4.8%) | <0.001 |
| Asthma | 37 (6.4%) | 81 (4.5%) | 0.061 |
| Pneumoconiosis | 4 (0.7%) | 13 (0.7%) | 0.951 |
| Cancer | 94 (16.3%) | 299 (16.5%) | 0.901 |
| Diabetes mellitus | 43 (7.4%) | 197 (10.9%) | 0.017 |
| Congestive heart failure | 46 (8.0%) | 115 (6.3%) | 0.176 |
| Autoimmune disease | 32 (5.5%) | 47 (2.6%) | <0.001 |
| HIV infection | 14 (2.9%) | 43 (2.4%) | 0.944 |
| Liver cirrhosis | 9 (1.6%) | 39 (2.2%) | 0.376 |
| Transplant | 9 (1.6%) | 9 (0.5%) | 0.022 |
| Chronic kidney disease | 9 (1.6%) | 11 (0.6%) | 0.037 |
| Steroid user | 46 (8.0%) | 113 (6.2%) | 0.146 |

Data are number (%).

Abbreviation: COPD, chronic obstructive pulmonary disease; TB, tuberculosis;

**Supplementary Table S2D.** Clinical characteristics of new episodes of *Mycobacterium abscessus* pulmonary infection and colonization in six hospitals

| **Characteristics** | **New Infection**  **(408 episodes)** | **New Colonization (1,059 episodes)** | **P-value** |
| --- | --- | --- | --- |
| **Age group** |  |  |  |
| Age <25 | 5 (1.2%) | 35 (3.3%) | 0.034 |
| Age 25~44 | 31 (7.6%) | 144 (13.6%) | 0.002 |
| Age 45~64 | 143 (35.0%) | 338 (31.9%) | 0.252 |
| Age >=65 | 229 (56.1%) | 542 (51.2%) | 0.089 |
| **Gender** |  |  |  |
| Male | 207 (50.7%) | 580 (54.8%) | 0.165 |
| **Comorbidity** |  |  |  |
| History of pulmonary TB | 108 (26.5%) | 242 (22.9%) | 0.145 |
| COPD | 39 (9.6%) | 89 (8.4%) | 0.483 |
| Bronchiectasis | 35 (8.6%) | 55 (5.2%) | 0.017 |
| Interstitial lung disease | 11 (2.7%) | 17 (1.6%) | 0.176 |
| Asthma | 12 (2.9%) | 30 (2.8%) | 0.911 |
| Pneumoconiosis | 1 (0.2%) | 5 (0.5%) | 0.549 |
| Cancer | 31 (7.6%) | 97 (9.2%) | 0.343 |
| Diabetes mellitus | 25 (6.1%) | 62 (5.9%) | 0.843 |
| Congestive heart failure | 15 (3.7%) | 33 (3.1%) | 0.589 |
| Autoimmune disease | 8 (2.0%) | 13 (1.2%) | 0.294 |
| HIV infection | 16 (3.9%) | 27 (2.5%) | 0.166 |
| Liver cirrhosis | 7 (1.7%) | 20 (1.9%) | 0.825 |
| Transplant | 4 (1.0%) | 11 (1.0%) | 0.921 |
| Chronic kidney disease | 4 (1.0%) | 9 (0.9%) | 0.811 |
| Steroid user | 15 (3.7%) | 24 (2.3%) | 0.136 |

Data are number (%).

Abbreviation: COPD, chronic obstructive pulmonary disease; TB, tuberculosis;

**Supplementary Table S2E.** Clinical characteristics of new episodes of *Mycobacterium kansasii* pulmonary infection and colonization in six hospitals

| **Characteristics** | **New Infection**  **(184 episodes)** | **New Colonization (403 episodes)** | **P-value** |
| --- | --- | --- | --- |
| **Age group** |  |  |  |
| Age <25 | 0 (0.0%) | 8 (2.0%) | 0.155 |
| Age 25~44 | 21 (11.4%) | 38 (9.4%) | 0.459 |
| Age 45~64 | 61 (33.1%) | 106 (26.3%) | 0.089 |
| Age >=65 | 102 (55.4%) | 251 (62.3%) | 0.984 |
| **Gender** |  |  |  |
| Male | 124 (67.4%) | 256 (63.5%) | 0.363 |
| **Comorbidity** |  |  |  |
| History of pulmonary TB | 62 (33.7%) | 76 (18.9%) | <0.001 |
| COPD | 28 (15.2%) | 58 (14.4%) | 0.793 |
| Bronchiectasis | 21 (11.4%) | 34 (8.4%) | 0.253 |
| Interstitial lung disease | 9 (4.9%) | 11 (2.7%) | 0.186 |
| Asthma | 8 (4.3%) | 9 (2.2%) | 0.164 |
| Pneumoconiosis | 3 (1.6%) | 1 (0.3%) | 0.102 |
| Cancer | 12 (6.5%) | 44 (10.9%) | 0.096 |
| Diabetes mellitus | 9 (4.9%) | 24 (6.0%) | 0.604 |
| Congestive heart failure | 8 (4.3%) | 15 (3.7%) | 0.717 |
| Autoimmune disease | 8 (4.3%) | 8 (2.0%) | 0.111 |
| HIV infection | 4 (2.2%) | 6 (1.5%) | 0.554 |
| Liver cirrhosis | 1 (0.5%) | 6 (1.5%) | 0.348 |
| Transplant | 1 (0.5%) | 5 (1.2%) | 0.449 |
| Chronic kidney disease | 0 (0.0%) | 2 (0.5%) | 0.592 |
| Steroid user | 8 (4.3%) | 15 (3.7%) | 0.717 |

Data are number (%).

Abbreviation: COPD, chronic obstructive pulmonary disease; TB, tuberculosis;

**Supplementary Table S3.** Prevalence of underlying comorbidity in new episodes of nontuberculous mycobacterial (NTM) pulmonary infection in different sexes

| **Characteristics** | **MALE**  **(N= 932)** | **FEMALE**  **(N= 742)** | ***p* value** |
| --- | --- | --- | --- |
| **Comorbidity** |  |  |  |
| History of pulmonary tuberculosis | 265 (28.4%) | 160 (21.6%) | 0.0014 |
| Chronic obstructive pulmonary disease | 252 (27.0%) | 140 (18.9%) | <0.0001 |
| Bronchiectasis | 115 (12.3%) | 146 (19.7%) | <0.0001 |
| Interstitial lung disease | 43 (4.6%) | 58 (7.8%) | 0.0068 |
| Asthma | 45 (4.8%) | 42 (5.7%) | 0.4466 |
| Pneumoconiosis | 13 (1.4%) | 0 (0.0%) | 0.0009 |
| Cancer | 98 (10.5%) | 56 (7.6%) | 0.0377 |
| Diabetes mellitus | 77 (8.3%) | 44 (5.9%) | 0.0684 |
| Congestive heart failure | 63 (6.8%) | 37 (5.0%) | 0.1298 |
| Autoimmune disease | 16 (1.7%) | 47 (6.3%) | <0.0001 |
| HIV infection | 29 (3.1%) | 19 (2.6%) | 0.5029 |
| Liver cirrhosis | 16 (1.7%) | 13 (1.8%) | 0.9561 |
| Transplant | 10 (1.1%) | 5 (0.7%) | 0.3936 |
| Chronic kidney disease | 4 (0.4%) | 9 (1.2%) | 0.0826 |
| Steroid user | 74 (7.9%) | 34 (4.6%) | 0.0061 |

**Supplementary Table S4A.** Radiographic findings and laboratory data of new episodes of nontuberculous mycobacterial infection and colonization in southern Taiwan

| **Characteristics** | **New Infection**  **(878 episodes)** | **New Colonization (3,114 episodes)** | **P-value** |
| --- | --- | --- | --- |
| **CXR pattern** |  |  |  |
| Fibocavitary | 251 (28.6%) |  |  |
| Nodular bronchiectatic | 627 (71.4%) |  |  |
| **CXR extent** |  |  |  |
| Focal | 228 (26.0%) |  |  |
| Multifocal | 650 (74.0%) |  |  |
| **Blood tests*** |  |  |  |
| Hemoglobin <12 g/dL | 152 (44.3%) | 503 (44.3%) | 0.990 |
| Platelet count <140 K/uL | 51 (14.9%) | 198 (17.4%) | 0.269 |
| Leukocyte <4000 or >10500 /uL | 83 (24.2%) | 330 (29.0%) | 0.081 |
| Segment >70% | 104 (73.8%) | 341 (64.2%) | 0.033 |
| C-reactive protein >5 mg/L | 198 (75.9%) | 660 (75.4%) | 0.886 |
| AST >40 U/L | 62 (19.6%) | 225 (21.4%) | 0.494 |
| ALT >40 U/L | 46 (13.8%) | 177 (15.9%) | 0.361 |
| Total bilirubin >1.0 mg/dL | 42 (23.6)% | 151 (26.9%) | 0.380 |
| Creatinine >1.4 mg/dL | 51 (14.7%) | 203 (17.6%) | 0.208 |

Data are number (%) or percentage.

Abbreviation: ALT, alanine transaminase; AST, aspartate transaminase;

* Data are the percentage of episodes with the characteristics among all tested episodes.

**Supplementary Table S4B.** Radiographic findings and laboratory data of new episodes of nontuberculous mycobacterial infection and colonization in northern Taiwan

| **Characteristics** | **New Infection**  **(796 episodes)** | **New Colonization (3,902 episodes)** | **P-value** |
| --- | --- | --- | --- |
| **CXR pattern** |  |  |  |
| Fibrocavitary | 266 (33.4%) |  |  |
| Nodular bronchiectatic | 530 (66.6%) |  |  |
| **CXR extent** |  |  |  |
| Focal | 247 (31.0%) |  |  |
| Multifocal | 549 (69.0%) |  |  |
| **Blood tests*** |  |  |  |
| Hemoglobin <12 g/dL | 280 (37.6%) | 989 (37.1%) | 0.808 |
| Platelet count <140 K/uL | 89 (12.0%) | 376 (14.1%) | 0.129 |
| Leukocyte <4000 or >10500 /uL | 216 (29.0%) | 656 (24.6%) | 0.015 |
| Segment >70% | 352 (50.1%) | 1099 (45.0%) | 0.017 |
| C-reactive protein >5 mg/L | 324 (67.8%) | 980 (63.4%) | 0.077 |
| AST >40 U/L | 122 (17.7%) | 391 (16.1%) | 0.330 |
| ALT >40 U/L | 92 (12.8%) | 345 (13.2%) | 0.761 |
| Total bilirubin >1.0 mg/dL | 104 (16.6%) | 414 (19.3%) | 0.135 |
| Creatinine >1.4 mg/dL | 74 (9.8%) | 308 (11.4%) | 0.225 |

Data are number (%) or percentage.

Abbreviation: ALT, alanine transaminase; AST, aspartate transaminase;

* Data are the percentage of episodes with the characteristics among all tested episodes.

**Supplementary Table S4C.** Radiographic findings and laboratory data of new episodes of *Mycobacterium avium*-*intracellulare* complex (MAC) pulmonary infection and colonization in six hospitals

| **Characteristics** | **New Infection**  **(577 episodes)** | **New Colonization (1,818 episodes)** | **P-value** |
| --- | --- | --- | --- |
| **CXR pattern** |  |  |  |
| Fibrocavitary | 165 (28.6%) |  |  |
| Nodular bronchiectatic | 412 (71.4%) |  |  |
| **CXR extent** |  |  |  |
| Focal | 142 (24.6%) |  |  |
| Multifocal | 435 (75.4%) |  |  |
| **Blood tests*** |  |  |  |
| Hemoglobin <12 g/dL | 226 (43.7%) | 535 (44.0%) | 0.925 |
| Platelet count <140 K/uL | 73 (14.1%) | 190 (15.6%) | 0.428 |
| Leukocyte <4000 or >10500 /uL | 158 (30.6%) | 349 (28.7%) | 0.430 |
| Segment >70% | 214 (56.3%) | 504 (52.8%) | 0.249 |
| C-reactive protein >5 mg/L | 254 (71.4%) | 564 (68.7%) | 0.364 |
| AST >40 U/L | 91 (19.0%) | 205 (18.4%) | 0.785 |
| ALT >40 U/L | 67 (13.3%) | 165 (13.7%) | 0.833 |
| Total bilirubin >1.0 mg/dL | 67 (18.5%) | 191 (22.2%) | 0.145 |
| Creatinine >1.4 mg/dL | 58 (11.0%) | 178 (14.3%) | 0.057 |

Data are number (%) or percentage.

Abbreviation: ALT, alanine transaminase; AST, aspartate transaminase;

* Data are the percentage of episodes with the characteristics among all tested episodes.

**Supplementary Table S4D.** Radiographic findings and laboratory data of new episodes of *Mycobacterium abscessus* pulmonary infection and colonization in six hospitals

| **Characteristics** | **New Infection**  **(408 episodes)** | **New Colonization ( 1,059 episodes)** | **P-value** |
| --- | --- | --- | --- |
| **CXR pattern** |  |  |  |
| Fibrocavitary | 107 (26.2%) |  |  |
| Nodular bronchiectatic | 301 (73.8%) |  |  |
| **CXR extent** |  |  |  |
| Focal | 124 (30.4%) |  |  |
| Multifocal | 284 (69.6%) |  |  |
| **Blood tests*** |  |  |  |
| Hemoglobin <12 g/dL | 58 (37.4%) | 156 (42.4%) | 0.291 |
| Platelet count <140 K/uL | 15 (9.7%) | 52 (14.1%) | 0.164 |
| Leukocyte <4000 or >10500 /uL | 50 (32.3%) | 103 (28.0%) | 0.327 |
| Segment >70% | 79 (53.4%) | 154 (45.4%) | 0.106 |
| C-reactive protein >5 mg/L | 77 (70.6%) | 146 (65.2%) | 0.320 |
| AST >40 U/L | 24 (16.2%) | 57 (16.6%) | 0.912 |
| ALT >40 U/L | 17 (11.1%) | 51 (13.9%) | 0.391 |
| Total bilirubin >1.0 mg/dL | 20 (15.0%) | 56 (17.8%) | 0.471 |
| Creatinine >1.4 mg/dL | 18 (11.5%) | 43 (11.5%) | 0.981 |

Data are number (%) or percentage.

Abbreviation: ALT, alanine transaminase; AST, aspartate transaminase;

* Data are the percentage of episodes with the characteristics among all tested episodes.

**Supplementary Table S4E.** Radiographic findings and laboratory data of new episodes of *Mycobacterium kansasii* pulmonary infection and colonization in six hospitals

| **Characteristics** | **New Infection**  **( 184 episodes)** | **New Colonization ( 403 episodes)** | **P-value** |
| --- | --- | --- | --- |
| **CXR pattern** |  |  |  |
| Fibocavitary | 78 (42.4%) |  |  |
| Nodular bronchiectatic | 106 (57.6%) |  |  |
| **CXR extent** |  |  |  |
| Focal | 39 (21.2%) |  |  |
| Multifocal | 145 (78.8%) |  |  |
| **Blood tests*** |  |  |  |
| Hemoglobin <12 g/dL | 28 (41.2%) | 61 (38.6%) | 0.717 |
| Platelet count <140 K/uL | 8 (11.8%) | 29 (18.4%) | 0.220 |
| Leukocyte <4000 or >10500 /uL | 18 (26.5%) | 63 (39.9%) | 0.054 |
| Segment >70% | 43 (63.2%) | 82 (55.8%) | 0.303 |
| C-reactive protein >5 mg/L | 36 (80.0%) | 68 (74.7%) | 0.495 |
| AST >40 U/L | 17 (25.8%) | 30 (20.8%) | 0.427 |
| ALT >40 U/L | 10 (15.2%) | 25 (15.8%) | 0.900 |
| Total bilirubin >1.0 mg/dL | 10 (15.9%) | 30 (22.4%) | 0.289 |
| Creatinine >1.4 mg/dL | 13 (18.3%) | 19 (11.8%) | 0.185 |

Data are number (%) or percentage.

Abbreviation: ALT, alanine transaminase; AST, aspartate transaminase;

* Data are the percentage of episodes with the characteristics among all tested episodes.

**Table S5. Independent risk factors of patients of pulmonary infection by nontuberculous mycobacteria in multivariate logistic regression analysis**

| **Characteristics** | **Odds Ratio (95% CI)** | **P-value** |
| --- | --- | --- |
| Location (southern vs. northern Taiwan) | 1.70 (1.49-1.94) | <0.001 |
| Age < 25 (yes vs. no) | 0.45(0.25-0.83) | 0.01 |
| Age between 25~45 (yes vs. no) | 0.69 (0.54-0.87) | 0.002 |
| Previous history of tuberculosis (yes vs. no) | 1.30 (1.12-1.51) | 0.001 |
| Chronic obstructive pulmonary disease (yes vs. no) | 1.24 (1.05-1.47) | 0.014 |
| Bronchiectasis (yes vs. no) | 1.79 (1.43-2.24) | <0.001 |
| Autoimmune (yes vs. no) | 1.79 (1.30-2.46) | 0.001 |
| Acquired immunodeficiency syndrome (yes vs. no) | 1.92 (1.22-3.02) | 0.005 |
| *M. avium*-*intracellulare* complex (yes vs. no) | 2.96 (2.41-3.65) | <0.001 |
| *M. abscessus* (yes vs. no) | 3.64 (2.81-4.71) | <0.001 |
| *M. kansasii* (yes vs. no) | 3.41 (2.78-4.19) | <0.001 |

* Each case was only counted his/her first episode, and we excluded those patients, who had more than one specimens isolated at the first day**.** We finally enrolled 7256 patients into this multivariate logistic regression analysis model

** All variables in **Table 2** except radiographic pattern and extent, and lab data were included in multivariate logistic regression analysis.
